# CDK9 inhibitors in multiple myeloma: a review of progress and perspectives

**DOI:** 10.1007/s12032-021-01636-1

**Published:** 2022-01-29

**Authors:** Jędrzej Borowczak, Krzysztof Szczerbowski, Navid Ahmadi, Łukasz Szylberg

**Affiliations:** 1 Department of Clinical Pathomorphology, Collegium Medicum in Bydgoszcz, Nicolaus Copernicus University in Torun, Bydgoszcz, Poland; 2 Department of Cardiothoracic Surgery, Royal Papworth Hospital, Cambridge, UK; 3 Department of Tumor Pathology and Pathomorphology, Oncology Centre-Prof. Franciszek Łukaszczyk Memorial Hospital, Bydgoszcz, Poland

**Keywords:** CDK9, Myeloma, Resistance, Synergism, p53, Bortezomib

## Abstract

Currently, multiple myeloma is not yet considered a curable disease. Despite the recent advances in therapy, the average patient lifespan is still unsatisfactory. Recently, CDK9 inhibitors emerged as a suitable agent to overcome resistance and prolong survival in patients with poor diagnoses. Downregulation of c-MYC, XIAP, Mcl-1 and restoration of p53 tumor-suppressive functions seems to play a key role in achieving clinical response. The applicability of the first generation of CDK9 inhibitors was limited due to relatively high toxicity, but the introduction of novel, highly selective drugs, seems to reduce the effects of off-target inhibition. CDK9 inhibitors were able to induce dose-dependent cytotoxicity in Doxorubicin-resistant, Lenalidomide-resistant and Bortezomib-resistant cell lines. They seem to be effective in cell lines with unfavorable prognostic factors, such as p53 deletion, *t*(4; 14) and *t*(14; 16). In preclinical trials, the application of CDK9 inhibitors led to tumor cells apoptosis, tumor growth inhibition and tumor mass reduction. Synergistic effects between CDK9 inhibitors and either Venetoclax, Bortezomib, Lenalidomide or Erlotinib have been proven and are awaiting verification in clinical trials. Although conclusions should be drawn with due care, obtained reports suggest that including CDK9 inhibitors into the current drug regimen may turn out to be beneficial, especially in poor prognosis patients.

## Introduction

Multiple myeloma (MM) is the second most common hematological malignancy characterized by monoclonal plasma cell growth leading to the production of non-functional immunoglobulins [1]. MM is characterized by over 138.000 cases per year worldwide and an approximately 2 per 100,000 incidence rate [2]. Multiple myeloma derives from monoclonal gammopathy of undetermined significance (MGUS) transformed plasma cells. Recent studies suggest that the early genetic changes leading to MGUS transformation are related to cyclin D protein dysregulation which can be observed in nearly 50% of cases [3]. The overexpressed cyclin D1 was connected with better chemotherapy response in newly diagnosed MM. MYC and RAS gene mutations are other common findings in multiple myeloma [4]. Interestingly, c-MYC expression is increased in myeloma cells in relation to MGUS thus suggesting it to be the key player in MGUS to MM transition [5]. Mcl-1 and Bcl-2 dysregulations are subsequent molecular changes enabling MM cells to escape apoptotic mechanisms and promote progression [6].

Due to the introduction of novel drugs, such as Bortezomib (BTZ), the estimated survival rate of MM patients improved significantly. For newly diagnosed patients receiving an autologous stem cell transplant (ASCT), the 3-year overall survival rate has increased from 45% in 1992–1998 to 80% in 2014–2016 [7]. However, acquired drug resistance results in limited long-term survival [1]. The outcomes are highly dependent on the presence of karyotype changes. Translocation of *t*(11:14) is deemed a favorable marker, while *t*(14:16) and *t*(14:20) are predictors of poor prognosis. Del (17.13) is another poor prognostic factor, bound with resistance to Bortezomib. It is presumably related to the loss of TP53 tumor-suppressing functions, which is also an independent prognostic factor [1, 8]. The advances and possibilities of their use in the treatment of multiple myeloma will be discussed later. As for now, the disease is treatable, but not yet curable [9].

### Cyclin-dependent kinases

**Fig. 1:**
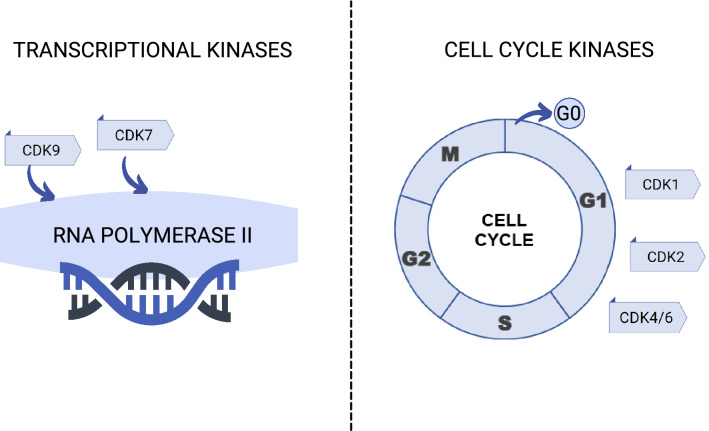
Role of main cyclin-dependent kinases

Cyclin-dependent kinases (CDK) are a family of enzymes regulating the cell cycle and transcription. Together with cyclins, another protein group, they form active complexes that control cell survival and proliferation [10]. Depending on their functions, CDKs can be divided into two main subgroups, namely transcriptional and cell cycle regulators. CDKs 1–6 and 14–18 control cell cycle, whereas CDKs 7–13 regulate transcription [11]. Recent studies have shown a potential clinical benefit of targeting certain proteins from the CDK family in multiple neoplasms [12]. (Fig. 1) CDK7 inhibitors are tested as single agents or in combination with fulvestrant in small cell lung cancer and breast cancer (NCT04247126). Several CDK 4/6 inhibitors, such as abemaciclib, palbociclib and ribociclib were recently approved by FDA and EMA in the treatment of HR+/HER2− mBC/ABC breast cancer. These drugs are currently ongoing in multiple clinical trials in other types of breast cancer as well as in other neoplasms such as head and neck squamous cell carcinoma and glioblastoma [13, 14].

CDK9 is a member of the transcriptional cyclin-dependent kinases family which can be found in two isoforms CDK9_42_ and CDK9_55_ [15]. Together with cyclins T1, T2a, and T2b it forms a Positive Transcription Elongation Factor (P-TEFb). Most of the cellular P-TEFb is inactive, sequestrated by the 7SK snRNA complex, but can be mobilized through BRD4 binding. Together, P-TEFb and BRD4 are capable of phosphorylating RNA pol II, sustaining transcription [15, 16]. Although CDK9_42_ and CDK9_55_ share the ability to phosphorylate RNA pol II there seems to be some difference between their function. Recent studies have shown a correlation between increased cell proliferation and upregulation of the CDK9_42_, whereas the CDK9_55_ seems not to have that relationship [17]. Furthermore, CDK9_55_ was suggested to take part in DNA repair mechanisms via the Ku70 associated pathway [18]. A similar variation in function was observed between CDK9 related cyclins. Cyclin T was deemed necessary for the differentiation of multiple cell lines including monocytes, lymphocytes and adipocytes [19]. Whereas Cyclin K was shown to be upregulated through p53 activation suggesting its role in DNA repair due to stress [20]. The role of the CDK9 in cancer pathogenesis is not fully established yet, but several studies proved it to be a poor prognostic factor in various cancers [12]. CDK9 was shown to take part in c-MYC oncogene activation and Mcl-1 and Bcl-2 protein overexpression. Since those proteins were proved to have an important role in the progression of hematological malignancies, CDK9 inhibitors are currently widely researched in those diseases [15].

## Novel CDK9 inhibitors in multiple myeloma

**Fig. 2:**
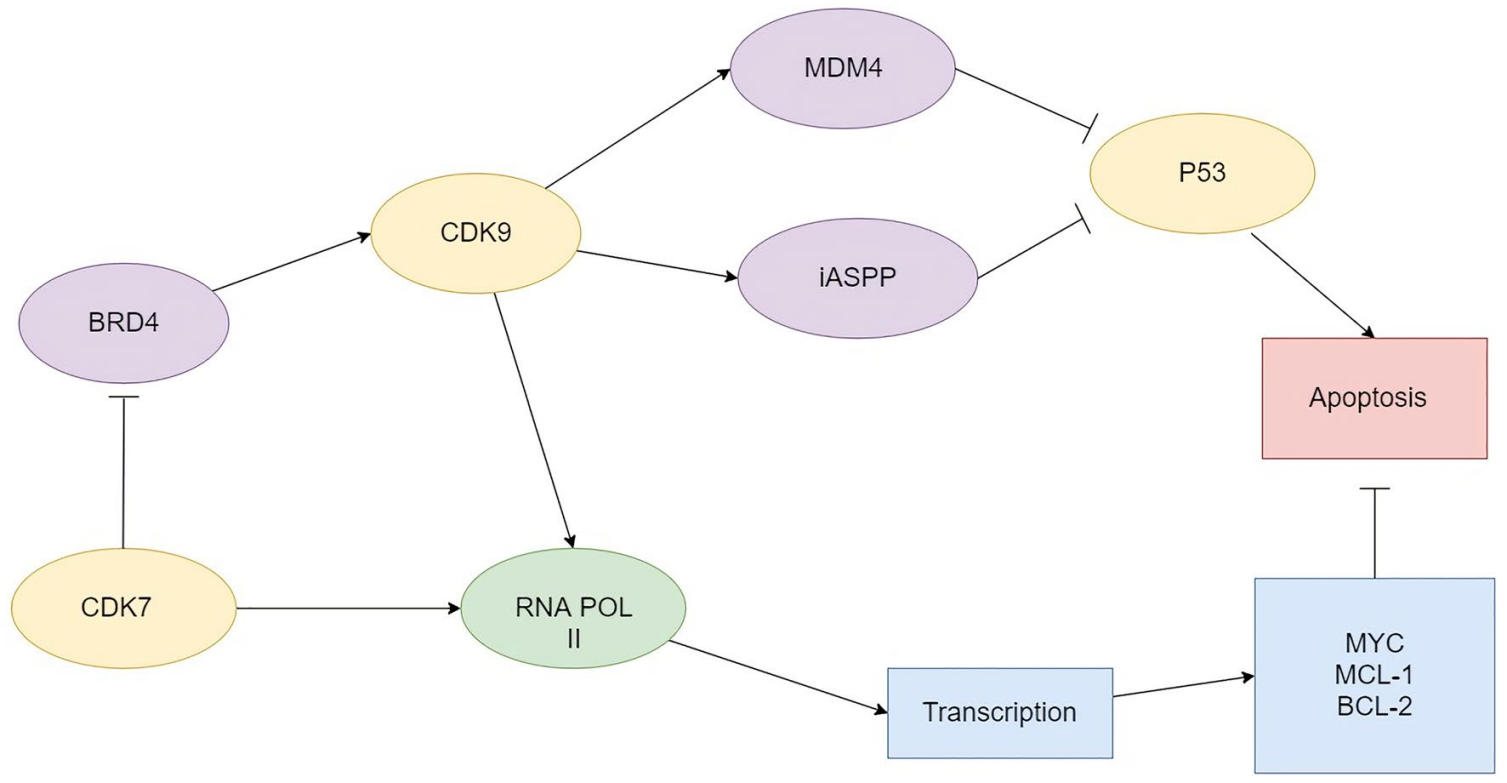
Role of CDK9 in transcription and apoptosis. *CDK* cyclin-dependent kinase, *BRD4* bromodomain containing 4, *MDM4* mouse double minute 4, *iASPP* inhibitor of apoptosis-stimulating protein of p53

CDK9 inhibitors recently gained more attention due to the application of Flavopiridol in breast cancer therapy and its synergistic effect with Trastuzumab [21, 22]. These drugs are currently undergoing thorough investigation in multiple hematological diseases (Table 1). Inhibition of CDK9 downregulates key metabolic pathways required for malignant cell survival and proliferation, for example, decreasing Mcl-1, XIAP and MYC expression [10]. Interestingly, p53 target genes function through CDK9-mediated transcription, while CDK9 inhibition downregulates p53 transcription and can increase the concentration of p53. The outcome depends on the degree of CDK9 blockade. Incomplete CDK9 blockade may trigger reactivation of residual CDK9 activity and overrun initial inhibition [10, 23]. Štětková et al. suggested that this effect can also be related to the inhibition of p53-opposing factors, such as mouse double minute 4 (MDM4) overexpression in tumors [24]. Moreover, CDK9 inhibitors downregulate the inhibitor of apoptosis-stimulating protein of p53 (iASPP), restoring p53 tumor-suppressing functions and opening a new perspective for the treatment of patients with loss of p53 function [25] (Fig. 2).

**Table 1:** Clinical trials of CDK9 inhibitors in hematologic malignancies

Drug	Neoplasm	Phase	clinicaltrial.gov
Dinaciclib	Chronic Lymphocytic Leukemia	III	NCT01580228
AT-7519	Chronic Lymphocytic Leukemia	II	NCT01627054
P276-00	Mantle cell lymphoma	II	NCT00843050
AZD-4573	Hematological malignancies	I/II	NCT04630756
Alvocidib/Flavopiridol	Chronic lymphocytic leukemia	II	NCT00464633
CYC065	Solid tumors or lymphomas	I	NCT02552953
Atuveciclib	Acute leukemia	I	NCT02345382
BAY-1251152	Hematological malignancies	I	NCT02745743
Voruciclib	Hematological malignancies	I (recruiting)	NCT03547115
GFH009	Hematological malignancies	I (not yet recruiting)	NCT04588922

Despite recent progress in MM treatment, most patients develop resistance to therapy. Immunoresistance and inevitable relapse seems to be among the urgent challenges [26]. Although some clinicians proposed that MM is curable cancer [27], others argue that these patients lose over 25 years of life when compared to a healthy population and still need continuous therapy, which makes the use of the word “curable” ungrounded [9].

The pathomechanism of resistance in MM is complex. Bone marrow stromal cells (BMSCs) can prevent apoptosis by adhesion-mediated drug resistance, upregulating the expression of anti-apoptotic Bcl-2 family proteins and promoting the autocrine loop to facilitate proliferation and progression [26, 28, 29]. CDK9 inhibitors can potentially induce apoptosis, overcome resistance in Melphalan-, Bortezomib- and Doxorubicin-resistant cell lines and resensitize the “survivor” cells to re-treatment [30–32].

Multiple novel CDK9 inhibitors are currently used as either single agents or co-therapeutics in multiple myeloma (Tables 2 and 3). The first generation of CDK9 inhibitors (Flavopiridol and Seliciclib) were pan-CDK inhibitors, prone to cause off-target toxicity. As the understanding of CDK9 biology grew, more selective CDK inhibitors were introduced. In the following part, we will discuss recent advances in the use of CDK9 inhibitors for multiple myeloma therapy.

**Table 2:** Clinical trials of CDK9 inhibitors in multiple myeloma

Drug	Selectivity	Clinical trials	Phase	Status	Study completion date
AZD-4573	CDK9	NCT03263637	I	Recruiting	N/A
P276-00	CDK9-T1, CDK4-D1 and CDK1-B	NCT00882063	I/II	Completed	May 2012
		NCT00547404	I	Withdrawn	July 2010
AT-7519 + Bortezomib	CDK1,2, 4, 5, 6 and 9	NCT01183949	I/II	Completed	March 2015
SNS-032	CDK2, 7, 9	NCT00446342	I	Completed	December 2009
RGB-286638	CDK 1–9	NCT01168882	I	Withdrawn (sponsor decision)	N/A
Dinaciclib	CDK1, 2, 5 and 9	NCT01096342	II	Completed	December 2012
		NCT00871663	I	Completed	October 2012
		NCT00871910	I	Completed	February 2010
		NCT01711528	I	Completed	November 2016
		NCT02684617	I	Terminated	N/A

**Table 3:** Preclinical trials of CDK9 inhibitors

Drug	Trial setup	Inhibited targets	Clinical effect
P276-00	MM xenograft; SCID murine model [33]	CDK9, Mcl-1; RNA polymerase II, cyclin T1	26% tumor mass reduction; 63% growth inhibition
	MM xenograft; SCID murine model [34]	CDK9, Cyclin D1, pRB, CDK4,	Tumor cell apoptosis; tumor growth arrest; 10% mice mass reduction
SLM-6 [35]	MM xenograft; SCID murine model	CDK9, c-Myc, cyclin D1, RNA polymerase II, c-Maf	60–80% MM cells apoptosis; tumor mass reduction; no signs of systemic toxicity;
AT751 [36]	MM xenograft; SCID murine model	CDK9, cyclin D1, cyclin A, cyclin B1, Mcl-1, XIAP	MM cells growth suppression; 45,5% longer overall survival time; tumor mass reduction;
AAP1742 [37]	In-vitro, MM cell lines	CDK9, Mcl-1, Bcl-2, XIAP, RNA pol II,	Apoptosis and growth arrest of MM cells
MC180295 [32]	MM xenograft; murine model	CDK9	Delayed sensitization; increase in the sub-G1 subpopulation; improved mouse survival; PD-L1 downregulation; downregulation of EMT transcription factors;
AZD-4573 [38]	In-vitro; cell line- and patient-derived xenograft models in-vivo	CDK9, Mcl-1, CD45 +	Regression of MML for all treated mice (> 125 days) and 55% tumor volume reduction; AML tumor growth inhibition
RGB-286638 [39]	MM xenograft murine model	Mcl-1; XIAP;	Induction of p53-independent apoptosis; reduced transcription in mutant-p53 MM cells; induction of apoptosis in MM cells lines with mutant-p53

*MM* multiple myeloma, *SCID* severe combined immunodeficiency, *EMT* epithelial-mesenchymal transition, *PD-L1* programmed death ligand-1, *AML* acute myeloid leukemia

### AZD-4573

Non-selective inhibitors act in an ATP-competitive manner; therefore, they tend to target multiple CDKs, limiting their use as therapeutic agents. Due to the lack of selectivity, it was unclear whether therapeutic effects in previous trials are related to CDK9 inhibition alone [40]. AZD-4573 is a novel, highly selective CDK9 inhibitor that binds to CDK9 in complex with cyclin T1 near the αC-helix of CDK9, with no direct interaction with the ATP binding site nor the ligand [41]. Although it can block other CDKs, the inhibitory effect is > 25-fold more selective for CDK9 (IC_50_ (μM)a [ATP] 5 mM < 0.004) over CDK1, CDK2, CDK4, CDK6, and CDK7 upon short-term treatment of MCF7 cells. AZD-4573 can limit the activity of GSK-3α, GSK-3β, Jnk1 and DYRK2, but in much higher concentration [38]. It has an estimated t_1/2_ of 1.6 h in humans [41].

AZD-4573 was successful in downregulating the phosphorylated RNA pol II, MCL-1 and Myc expression with no effect on the total RNA pol II levels [38]. Furthermore, in combination with ARV825, bromodomain proteolysis targeting chimeric molecule (PROTAC), AZD-4573 showed further decrease in MCL-1, Myc, RNA pol II, BRD2, BRD3 and BRD4 expression. A higher apoptosis rate was observed in MM cell lines treated with both drugs in comparison to monotherapy. A synergistic effect has been shown in in-vivo MM xenograft models with no significant side effects, apart from minor weight loss (< 10%) [42]. AZD-4573 was able to cause apoptosis in T-cell lymphoma and AML xenograft models, showing the synergistic effect with Venetoclax [38]. According to Su-Lin Lim et al. study AZD-4573 can inhibit the proliferation of multiple myeloma cell lines in-vitro, including Bortezomib- and Lenalidomide-resistant cell lines [42].

Despite promising anti-cancer activity in-vivo and in-vitro, and high selectivity, there are currently no reports from clinical trials that can validate the effect in humans. AZD-4573 was recently undergoing a phase I clinical trial in relapsed/refractory hematologic malignancies (NCT03263637). Although the study ended in September 2020, the results have not been posted yet.

### MC180295

In the pursuit of epigenetic drugs that can reverse the silencing of tumor suppressor genes in cancer, Zhang et al. investigated the role of CDK9 in gene silencing in cancers. They discovered MC180295, a potent CDK9 inhibitor that binds to the C-terminal part of CDK9 through the norbornyl group, whose selectivity results from the subtle structural variation in the active site. The drug's potency toward CDK9 (IC_50_ = 5 nM) is over 22-fold stronger than for other CDKs. It also seems to downregulate GSK-3a and GSK-3b via non-gene activation mechanisms. The inhibition of CDK9, induced by MC180295 led to dephosphorylation of BRG1, which contributed to the restoration of tumor suppressor gene expression [32].

MC180295 inhibits the proliferation of numerous multiple myeloma cell lines. Even though MC180295 showed higher selectivity toward CDK9 than AZD-4573, it was not as potent [42]. MC180295 downregulated Myc and Mcl-1 in mantle cell lymphoma cell lines, as well as in Ibrutinib- and Venetoclax-resistant cell lines. A synergistic effect of Venetoclax and MC180295 was observed [43]. When compared to SNS-032 in NSG mice injected with SW48 cells, MC180295 slowed tumor growth slower and improved mouse survival without causing overt toxicity [32].

The broad MC180295 anti-cancer activity in-vivo and in-vitro seems promising, although the lack of toxicity and higher selectivity goes hand in hand with relatively lower potency toward CDK9 when compared to AZD-4573.

### SLM-6

Sangivamycin was originally isolated from Streptomyces rimosus, and subsequently tested in phase I clinical trial in the 1960s [44]. It showed anti-tumor and anti-retroviral properties, safety in humans; however no further studies were conducted [35, 44]. Sangivamycin-Like Molecules (SLM) are nucleoside analogs of sangivamycin, which possess the same anti-tumor properties and were previously tested in preclinical models of colon cancer to overcome hypoxia-induced resistance to apoptosis [45]. Recently, Dolloff et al. reported that MM cells are sensitive to SLMs and identified SLM-6 as a lead compound with good tolerability and the most activity to inhibit growth and induce apoptosis of MM tumors [35].

SLM-6 inhibits phosphorylation of CDK9, critical to the kinase activity of P-TEFb, with preference to 55-kDa isoform of CDK9 [35, 46]. Unlike Flavopiridol, SLM-6 did not affect the phosphorylation of RNA polymerase II at Ser5, a CDK7 specific site. However, it was found to bind an autophosphorylation site of CDK9 at Thr186, a place critical to the kinase activity of P-TEFb [46]. in-vitro analysis showed that SLM-6 inhibits CDK9/cyclin K and CDK9/cyclin T1 with IC_50_’s of 280 nmol/L and 133 nmol/L, respectively. SLM-6 inhibits CDK1 and CDK2 (both IC_50_’s < 300 nmol/L), but only its activity against CDK9 induced MM cell death. The effect was similar when the authors treated MM cells with various CDKs inhibitors. Only the drugs with activity toward CDK9 were capable of downregulating c-Myc, c-Maf and cyclin D1 in RPMI-8226 cells suggesting the pivotal role of CDK9 inhibition in SLMs anti-MM activity. Another sangivamycin analog, SM-3, was able to induce dose-dependent apoptosis of MM cells but did not affect cell lines from other types of tumors. MM cells turned out to be more sensitive to SLM3 than to other nucleoside analogs, namely 5′-fluorouracil, gemcitabine, and cladribine. That resulted in a rapid reduction of MM cells viability, measured histologically [35].

In in-vivo studies, SLM-6 significantly reduced the size of MM tumors, while Flavopiridol showed no anti-MM activity at a dose 10 times higher than SLM-6 in the same model. The repeated dosing of SLM-6 in immunocompetent mice showed no signs of systemic toxicity and no effects on normal hematopoiesis, aside from modest thrombocytopenia. It may be the effect of direct inhibition of CDK9 in MM cells but not in any other cell lines. SLM-6 and bortezomib showed an additive therapeutic effect in NCI-H929 and CD138+ patients bone marrow cells. The combination of both agents was more effective in reducing MM cell viability than either of the drugs alone [35]. SLM-6 showed promising anti-cancer properties in-vivo and in-vitro, but to our best knowledge, there were no more reports regarding the use of SLMs in cancer studies.

### AAP1742

AAP1742 is an analog of CAN 508 discovered in the library of arylazo-3,5-diaminopyrazoles that is active in RPMI-8226 MM cell lines [37]. Although the compound acts primarily through inhibition of CDK9 (IC_50_ = 0.28 µM) it shows activity toward other CDKs (CDK2 IC_50_ = 0.549; CDK4 IC_50_ = 0.454), but in higher concentrations. It decreases the phosphorylation of RNA polymerase II and induces MM cells apoptosis by downregulating anti-apoptotic proteins Mcl-1, Bcl-2, and XIAP in a dose- and time-dependent manner.

The treatment of RPMI-8226 cells with AAP1742 induced suppression of proliferation and apoptosis at 10 lM concentration. After 24 h of treatment with 40 IM dose, Bcl-2 mRNA level decreased to 10% of the control, and Mcl-1 mRNA to 62% of the control. Mcl-1 downregulation was considered the event that initiated apoptosis in treated MM cells, while the cytotoxic activity of AAP1742 was attributed to cellular inhibition of CDK9 [37].

### AT-7519

AT-7519 is an ATP-competitive multi-CDK inhibitor with potent activity toward CDK1,CDK2,CDK4, CDK6,and CDK9 with IC_50_ values of 210,47,100,13,170, and < 10 nmol/L, respectively. It shows the selectivity for CDK9 and blocks RNA polymerase II phosphorylation, a CDK7/9 substrate, and glycogen synthase kinase 3β (GSK-3β) phosphorylation [36]. In-vitro and in-vivo studies showed its cytotoxicity toward MM cells, associated with in-vivo tumor growth inhibition and prolonged survival of mice. MM cell death occurred through the dephosphorylation of RNA pol II, which resulted in the inhibition of transcription [36]. AT-7519 anti-tumor properties were independent of p53 expression, while the drug was effective against HT29 and MDA-MB-468 cell lines expressing a mutant form of p53 [47].

Dose-dependent cytotoxicity of AT-7519 was determined in MM cell lines sensitive and resistant to Doxorubicin and Melphalan. Moreover, AT7951 partially overcomes the proliferative effects of bone marrow stromal cells (BMSCs), IL-6 and IGF-1, reducing resistance to Doxorubicin and Bortezomib. Prolonged exposure of MM cells to AT-7519 did not show additional cytotoxicity, suggesting maximum effect at 48 h. Starting from 2 h after the first dose of AT7591, Bcl-2 family proteins, cyclin D1, cyclin A, and cyclin B1 were downregulated. Moreover, AT-7519 did not induce cytotoxicity in peripheral blood mononuclear cells from five healthy volunteers [36, 48]. In mice, AT-7519 inhibited tumor growth when compared with controls (*P* < 0.05). The median overall survival of animals treated was significantly prolonged (39 days vs. 27.50 days respectively) [36].

AZT7519 was recently tested in combination with Bortezomib in patients with previously treated multiple myeloma [49]. The treatment was well-tolerated, and the maximum doses for both AZT7519 and Bortezomib were achieved (21 mg/m^2^ and 1.3 mg/m^2^, respectively). No significant efficacy was observed after treatment with AT7519M alone, but the combination of AT7519M with Bortezomib resulted in significant rate (33% ≥ partial remission) responses.

### P276-00

P276-00 is a flavone that arrests cells in the G1/S phase of the cell cycle. It shows selectivity toward inhibiting CDK9-T1, CDK-4-D1 and CDK1-B with IC_50_ values at 20 nM, 63 nM and 79 nM, respectively [33, 50]. It competes with ATP in the active site of CDKs causing either cell cycle arrest or apoptosis, but its efficacy is dose-dependent and cell-type dependent [50]. P276-00 acts mainly through inhibition of CDK9-T1, affecting primarily transcription of mRNA with short half-lives, such as Mcl-1 [33].

P276-00 inhibits tumor cell growth in culture 2 to 3 times stronger than Flavopiridol because of its higher selectivity toward CDK9. Hence, it’s less toxic than Flavopiridol but remains more potent in inhibiting tumor cell growth [33]. The treatment of myeloma cell lines with P276-00 caused transcription inhibition and a significant decline in Mcl-1 protein levels prior to MM cells death [33, 34]. P276-00-induced downregulation of Mcl-1 seems to switch the balance toward apoptosis, overcoming programmed cell death evasion in MM cells [33]. Treating cell lines for 3 h and 6 h resulted in a rapid, time and dose-dependent decrease in CDK9 and Mcl-1 expression. Other proteins from the Bcl-2 family and cyclin D1 with longer half-lives were significantly downregulated at the 24 h time-point. An increase in PARP cleavage and caspase-3 activity suggested the activation of apoptotic pathways [33, 34, 51]. An anti-MM synergistic effect of P276-00 and Bortezomib was observed in-vitro at a wide range of tested concentrations. P276-00 overcomes the growth and survival stimulation mediated by cytokines and bone marrow stem cells, alleviating the resistance to Bortezomib [34]. However, cyclin D1 overexpression may render the response to P276-00 therapy by making the MM cells more responsive to proliferative stimuli [34]. A synergistic effect of P276-00 and Doxorubicin was also reported in non-small cell lung carcinoma [42].

To confirm the in-vivo activity, P276-00 was administered intraperitoneally into RPMI-8226 xenograft for 15 days and reached the growth inhibition of 63% [33]. Reduction of the tumor mass and significant survival benefit in mice, compared to the control group, was observed after 30 days of P276-00 administration [34]. P276-00 was tested in phase I/II clinical trials to assess the safety and efficacy in patients with refractory multiple myeloma, but the results have not been published (Table 2).

P276-00 affects transcription of short half-live proteins, switching the in-cell balance toward apoptosis. The drug showed effectiveness in-vitro, in-vivo and enhanced the efficacy of Bortezomib, but the overexpression of cyclin D1 and other pro-apoptotic proteins may render its activity.

### RGB-286638

RGB-286638 is a non-selective CDK inhibitor with activity against CDK 1, 2, 3, 4, 5, 6, 7, 9 and has the highest potency toward CDK9 (IC_50_ = 1 nM). It has shown the ability to downregulate other serine-threonine and tyrosine kinases, such as JAK2, AMPK, TAK1, MEK 1 and GSK-3β [39].

RGB-286638 can effectively inhibit the transcription to total blockage after 24 h of exposition of in-vitro multiple myeloma cell lines. It inhibits both RNA and DNA, downregulating their synthesis by 50% and 60% respectively. Furthermore, accumulation of p53, MM associated mi-RNAs and NAD/NADH reduction was observed after application of RGB-286638. Treatment after 12 h and 24 h caused an increase in apoptosis of MM cells by 25% and 45%, respectively [39]. in-vivo examination showed significant multiple myeloma growth suppression and improved survival time in SCID mice (43 days vs 24 days in the control group). It also triggered dose-dependent cytotoxicity in Melphalan-resistant, Doxorubicin-resistant and steroid-resistant MM cells [52].

In phase I clinical trials RGB-286638 treatment resulted in stabilization of the disease by up to 14 months. However, some side effects emerged during the treatment, with the most significant being hypotension, tachycardia, troponin T and liver enzyme elevation. This led to recommended administration for phase II is suggested to be 120 mg/d for 5 days every 28 days [53].

### Dinaciclib

Dinaciclib interacts with acetyl-lysine recognition sites of bromodomains, primarily inhibiting CDK1, CDK2, CDK5, and CDK9 ((IC_50_ = 3, 1, 1, and 4 nM, respectively). Its high selectivity is probably associated with the binding interactions in the ATP site of CDKs [54].

In phase I clinical trial for patients with advanced malignancies, Dinaciclib suppressed the proliferation of stimulated lymphocytes and reduced Rb phosphorylation. Inhibition of CDK9 blocked the transcription of both *CCND1* and *hDM2*, leading to a reduction in cyclin D1 and increased p53 expression [55]. Dinaciclib enhanced the response to Doxorubicin in RPMI-8226 MM cells [56].

Dinaciclib, as a single agent led to a prolonged remission in 3 out of 27 patients (11%), and minimal response in 2 patients with relapsed MM. The overall response in refractory/relapsed multiple myeloma was 18.5% and was the highest in the patients treated with a 40 mg/m^2^ dose. The most common side effects were diarrhea (87%), fatigue (67%), thrombocytopenia (60%), and nausea (53%), but the treatment was overall well-tolerated [57].

Ghia et al. reported results of the only phase III study regarding the use of Dinaciclib when compared with Ofatumumab, an anti-CD20 antibody, in 44 patients with chronic lymphocytic leukemia (CLL) resistant to either fludarabine or chemoimmunotherapy [58]. Even though the patients assigned to the Dinaciclib group had more advanced disease (Rai stage IV 65% vs. 31,8%) compared to the Ofatumumab group, the Dinaciclib group achieved longer median PFS (13.7 vs. 5.9 months), longer OS (21.2 vs. 16.7 months) and higher ORR (40% vs. 8.3%). Interestingly, these differences increased significantly in patients with p53 deletion (median PFS 17.2 vs. 2.4 months; median OS 21.2 vs. 5.4 months), suggesting that CDK9 inhibitors might be beneficial for patients with refractory/relapsed disease and unfavorable in cytogenetic changes (Table 4).

**Table 4:** Clinical effects of Dinaciclib and Ofatumumab in 44 patients with chronic lymphocytic leukemia [58]

Drug	Medium PFS (months)		Medium OS (months)		ORR	
	Overall	P53 deletion	Overall	P53 deletion	Overall	P53 deletion
Dinaciclib	13.7	17.2	21.2	21.2	8/20 (40%)	N/A
Ofatumumab	5.9	2.4	16.7	5.4	2/24 (8.3%)	N/A

*PFS* progression-free survival, *OS* overall survival, *ORR* overall response rate

*The deletion of p53 was present in seven patients

Dinaciclib is currently under investigation in combined therapy with Bortezomib and Dexamethasone in the treatment of relapsed multiple myeloma (NCT01096342). It can potentially reduce exposure to cytotoxic chemotherapy and minimize side effects, by enhancing the activity of other drugs, such as Doxorubicin and Bortezomib [55].

### Seliciclib

Seliciclib is a roscovitine derivative, multipotent, ATP-competitive pan-CDK inhibitor with most activity against CDK2, CDK7 and CDK9 (IC_50_ = 0.1, 0.36 and 0.81 μm, respectively). Seliciclib can effectively kill MM cells in-vitro even with added protective factors such as Interleukin 6, VEGF and IGF-1. It showed an anti-tumor effect in multiple neoplastic cell lines and xenografts including non-small cell lung cancer, hepatocellular carcinoma and multiple myeloma [51]. The effect of 8 h Seliciclib infusion persisted up to 72 h, reducing the three different cell lines by a minimum of 50% and a reduction in Mcl-1 level was observed. These changes were mainly obtained via transcription inhibition thus suggesting the key role of CDK9 and CDK7 inhibition in the process.

Seliciclib might be a potent additional treatment to Bortezomib and Doxorubicin-based MM protocols due to the synergistic effect [59]. Zhang et al. revealed that “survivors” of CDK9 inhibition were also more sensitive to re-treatment, which may prove crucial in case of recurrence and long-term therapy [32].

### SNS-032

SNS-032 was previously described as a selective CDK2 inhibitor that possesses anti-tumor activity in animal models. Subsequent research revealed that it possesses the greatest potency toward CDK9 (IC_50_ = 4 nM) and weaker activity toward other kinases such as CDK2, CDK7 and GSK-3α (IC_50_ 38–48 nM, 62 nM and 230 nM, respectively) [60]. It inhibits phosphorylation of mTOR proteins, completely blocking the activity of mTORC1 and mTORC2 in HL-60 and KG-1 cells (IC_50_ = 200 and 400 nM), achieving a slight degradation of mTOR expression [61].

In RPMI-8226 MM cells SNS-032 transiently inhibited transcription and decreased the concentration of VEGF, XIAP and Mcl-1 transcripts within 2 h after infusion. Evaluation of CDK9 inhibition and PARP cleavage established a temporal association between CDK inhibition, downregulation of survival proteins, and apoptosis. In human plasma, SNS-032 kept its anti-MM activity and remained fivefold more potent than Flavopiridol [60]. H929 MM cells co-cultured with the bone marrow stromal cell line HS-5 showed resistance to SNS-032, which suggests that bone marrow stroma may play a pivotal role in the development of primary resistance to CDK9-targeted treatment. in-vitro exposure of patient-derived MM cells showed that SNS-032 induces apoptosis of CD138+ cells, but it's only mildly toxic to CD138- MM population and does not prevent the formation of CD34+ colonies derived from normal bone marrow [62].

SNS-032 was examined in phase I clinical trial (NCT00446342) in patients with advanced CLL and MM. Dose-limiting toxicities were not observed, while maximum-tolerated dose was not established due to the early closure of the study. In the MM group, 78% of patients experienced grade 3 to 4 neutropenia, thrombocytopenia or anemia. Other grades 3 to 4 adverse events were sporadic. The most common grade 1 to 2 adverse were nausea, vomiting, constipation, and diarrhea. The treatment was well-tolerated, but the efficacy was limited. As all patients in this study had two or more prior therapies, a better clinical response may be observed in the earlier-stage disease [63].

### Flavopiridol

Flavopiridol (Alvocidib) is a flavonoid alkaloid and the first pan-CDK inhibitor to enter clinical trials [64]. Its anti-cancer activity was originally attributed to its ability to induce cell cycle arrest at G1 and G2/M checkpoints through ATP-competitive inhibition of CDK1 and CDK4/6. Later it was found to be most effective against CDK7 and CDK9 (IC_50_ < 300 nM), but also able to inhibit both EGFR and PKA kinases (IC_50_ 21 and 122 µM, respectively) [65].

Flavopiridol downregulated the expression of anti-apoptotic proteins in ANBL-6, ARP1 and RPMI-8226 MM cells lines in-vitro. The decrease in Mcl-1, Bcl-XL and XIAP correlated with early apoptosis of MM cells, but the effect differed in various cell lines. Flavopiridol induced rapid apoptosis of MM cell lines, but Mcl-1 overexpression was able to limit Flavopiridol-induced cell death [66].

In phase II clinical trials of relapsed/refractory multiple myeloma flavopiridol showed no indication of anti-myeloma effects in any patient. The subsequent in-vitro study showed that although significant anti-myeloma effects were noted after 12 h to 24 h, no response was observed after 4 h of exposure. The results were then confirmed in another phase I clinical study [67]. Flavopiridol turned out to be unable to cause long-term anti-myeloma effects [68].

Recently, attempts to use Flavopiridol in MM have been resumed. Zhou et al. reported that Flavopiridol enhanced the efficacy of Venetoclax in MM cell lines that were primarily less responsive or unresponsive to Venetoclax-induced apoptosis. The synergistic effect was present in either U266, H929 and RPMI-8226 cell lines, as well as multiple other cell lines with unfavorable karyotypes [del 13, del 17p, *t*(11; 14)]. The combination has the potential to overcome MM-related and microenvironment-driven drug resistance by downregulating MCL-1 and upregulating BIM, proteins mediating resistance to Venetoclax. In both NOD/SCID-ɣ and immunocompetent mice Flavopiridol achieved longer survival than mice treated with Venetoclax (79 vs 63 days and 69 vs 49 days, respectively) [69].

While further trials with Flavopiridol as a single drug in MM seems inexpedient, the ability of CDK9 inhibitors to overcome resistance to therapy is well-grounded in literature [6, 28, 30, 66, 70]. In this case, Flavopiridol may prove effective, but it's being replaced by more selective drugs.

## Potential synergistic combinations with CDK9 inhibitors in multiple myeloma

Most of the reports regarding the use of CDK9 inhibitors in MM pertain to refractory or relapsed patients, in which previous treatment regimens turned out to be ineffective. In those settings, CDK9 inhibitors were primarily examined as co-therapeutics to alleviate the resistance to other drugs and enhance anti-tumor properties. Therefore, not much data are available regarding the clinical outcomes in patients with better prognosis. Furthermore, accurate safe doses, therapeutic doses, bioavailability and pharmacokinetics of individual drugs are still not clearly determined. Nonetheless, many authors suggest that CDK9 inhibitors may complement current treatment regimens and will be discussed below (Table 5).

**Table 5:** Potential combinations of CDK9 inhibitors with other agents [71]

Drug	Potential synergy	Mechanism of synergy/clinical effect	Type of effect
P276-00 [33, 51]; SLM-6 [35], AT-7519 [36]; Roscovitine [72]; Seliciclib [59]; Flavopiridol	Bortezomib, Carfilzomib	Cyclins and CDKs are substrates of proteasomes, which accumulation may lead to resistance to therapy; CDK9 inhibitors prevent drug resistance, obstruct the accumulation of anti-apoptotic proteins, diminish proteasomal protein degradation, promote cancer cells apoptosis and activate alternate apoptotic signaling cascades [34]	Additive/synergistic
P276-00 [73]; Dinaciclib [56]; Roscovitine[74]	Doxorubicin	Inhibition of doxorubicin-induced chemoresistance, involving reduction of (LPS)-induced NF-kB [75], inflammatory genes transcription[76] and TNF expression [77]	Synergistic
Flavopiridol [69]; Dinaciclib [78]; AZD-4573 [38]	BH-3 mimetics: Venetoclax; [79]	CDK9 decreases the transcription of Bcl-2 family proteins and upregulated BH-3 proteins expression; BH-3 mimetics block remaining Bcl-2 proteins activity, leading to apoptosis	Synergistic
SNS-032; Dinaciclib [80]; AZD-4573, MC180295 [42]	BET inhibitors; OTX015; ARV825, CPI-203 [81]	Targets of both drugs are positive regulators of P-TEFb; inhibition of transcription and tumors growth; cell cycle arrest, increased in MM cells apoptosis; c-MYC transcription inhibition	Synergistic
Dinaciclib [82]; Flavopiridol [83]	Ofatumumab, Rituximab, Cyclophosphamide	Mechanism remains unclear	Not defined
PHA-767491 [84]	Erlotinib—inhibitor EGFR	PHA-767491 overcomes the resistance to EGFR- based therapy; induction of apoptosis, G2-M cell cycle arrest, inhibition of DNA replication	Synergistic
Inhibition of CKIα, CDK7 and CDK9 [85]	Lenalidomide	Leukemia cells apoptosis by triggering DNA repair response and augmenting p53 activation; p53 stabilization; preservation of hematopoiesis	Synergistic

Bortezomib, a proteasome inhibitor, has revolutionized the treatment of MM, but despite its high initial response rate, Bortezomib loses efficacy over time [35]. Dai et al. suggested that a combination of Flavopiridol and Bortezomib acts synergistically through induction of mitochondrial damage, caspase activation, and apoptosis [72]. CDK inhibitors downregulate the transcription, reducing the number of anti-apoptotic proteins, while proteasome inhibition blocks the degradation of pro-apoptotic proteins. Hence, the combination of both drugs changes the intracellular balance to favor apoptosis. P276-00 was tested together with Bortezomib in myeloma cells and showed marked synergism [34], which coincides with the results of SLM-6 [86] and Dinaciclib [87]. The combination of Doxorubicin, Bortezomib and either P276-00 [34] or Seliciclib [59] were also deemed effective. Zhang et al. showed that Mcl-1 was upregulated in all tested MM lines, including the Bortezomib-resistant lines. Moreover, Mcl-1 overexpression significantly reduced Bortezomib cytotoxicity, indicating a functional role for Mcl-1 in Bortezomib resistance. CDK9 inhibition substantially potentiated the susceptibility of Bortezomib-resistant cells to both proteasome inhibitors and BH-3 mimetics [30]. On the other hand, Zabihi et al. study showed no significant enhancing effect of AT-7519 together with Bortezomib in KG-1 cells, suggesting that CDK9 inhibitors do not act by the activation of the proteasome pathway [88].

Venetoclax is a BH-3-mimetic that blocks the Bcl-2 protein, leading to cell apoptosis [89]. Voruciclib [90], A‐1467729 and A‐1592668 [79] were recently proven synergistic with Venetoclax via CDK9 inhibition. Treatment of mice with A-1592668, a selective CDK9 inhibitor, led to a significant increase in survival (median survival 24.5 days, *P* < 0.0001) compared to the control group (median survival 13.5 days). There was no significant weight loss, and the decrease in lymphocyte burden did not impact hemoglobin, neutrophil, or platelet counts. Venetoclax was substantially less active and did not provide any survival benefit. However, co-treatment of mouse lymphoma #4242 cell line tumors in-vitro with A-1592668 and Venetoclax extended the median survival from 30.5 to 41 days [91]. Voruciclib was reported to downregulate Mcl-1 and c-Myc, enhancing Venetoclax activity in AML models. However, the effect is transient and the drug needs to be administered repeatedly [90]. Similarly, AZD-4573 together with Venetoclax achieved prolonged regressions in 100% of treated mice, with all eight mice remaining tumor-free till day 63. The only notable side-effect was minimal bodyweight loss, suggesting that the combination was well-tolerated [38].

BRD4 is a member of the human BET protein family that binds acetylated histones during mitosis to maintain chromatin structure and ensure early re-initiation of transcription after mitosis [92]. BRD4 recruits P-TEFb and promotes the elongation of transcription. When used together, CDK9 and BRD4 inhibitors impede transcription of anti-apoptotic genes and c-MYC oncogene, suppressing tumor proliferation. Combination of ARV825 and AZD-4573 caused apoptosis of 67% of KMS11 cells and 71% of RPMI-8226 cells, significantly slowing MM tumor growth (*P* < 0.001) [42].

A synergy between CPI-203, a novel bromodomain inhibitor, and either Bortezomib or Lenalidomide was also observed [86, 87]. Lenalidomide contributes to overcoming resistance to Bortezomib via inhibition of IRF4, which leads to MYC downregulation [93]. CPI-203 represses MYC gene transcription and has a cytostatic effect on MCL cells in-vivo, while the cytotoxicity in peripheral blood from healthy donors was below 25%, indicating the drug’s selectivity. Lenalidomide alone partially reduced MYC and RF4 expression, but together with CPI-203 the expression of genes was almost completely abrogated. The combination of CPI-203 and lenalidomide induces programmed cell death in MCL, inhibits the growth of bortezomib-resistant cells in-vivo and reduces tumor volume [93]. While this combination has not been tested in multiple myeloma models Minzel et al. [85] showed that CDIα inhibition, which co-targets CDK7/9, underlines the therapeutic effect of lenalidomide in a pre-leukemia syndrome through p53 activation and stabilization [85]. Since the synergies between BET inhibitors and CDK9 inhibitors, as well as BET inhibitors and lenalidomide were confirmed, the addition of CDK9 inhibitors to standard lenalidomide-based therapy could prove beneficial.

### CDK9 degradation or CDK9 inhibition?

Currently, all CDK9 inhibitors that have advanced to the second phase of clinical trials are non-selective, reversible and require continuous target occupancy to maintain CDK9 inhibition [15, 94]. As those agents bind to the CDK9/cyclinT1 complex in an ATP-competitive manner, the CDK9 blockade may be prone to be overrun by residual CDK9 activity, limiting their clinical effectiveness [10, 12, 15, 23]. Due to the lack of human clinical trials with selective CDK9 inhibitors and the off-target toxicity of the first generation CDK9 inhibitors, an alternative method to blocking the CDK9 activity was sought. Recently, the degradation of CDK9 has been suggested as an alternative to CDK9 inhibition [15, 94–97]. CDK9, as an endogenous protein is stabilized by a chaperone pathway, which helps in forming a stable cyclin T1/CDK9 complex. The excess of CDK9 becomes very unstable and is rapidly degraded by the proteasome [95]. Robb et al. demonstrated that chemical degradation of CDK9 in HCT116 can be successfully induced by proteolysis targeting chimera (PROTAC) [96].

The PROTACs are bivalent chemical protein degraders that link specific endogenous proteins with a component of E3 ubiquitin ligase. In this way, the protein is polyubiquitinated and degraded [98]. Ubiquitination is associated with the functionality of the CRBN gene, whose product is a receptor of E3 ubiquitin ligase; hence, CRBN expression may affect the therapeutic effectiveness of protein degraders [94, 99]. This strategy seems promising, especially in the degradation of CDK 9–13, which are not associated with the cell cycle [100].

Olson et al. reported that THAL-SNS-032, a selective CDK9 degrader, together with NVP-2, a CDK9 inhibitor, induced rapid degradation of CDK9 without affecting the levels of other CDKs [94]. THAL-SNS-032 inhibited proliferation of MOLT4 cells at lower concentrations (IC_50_ = 50 nM) than SNS-032 (IC_50_ = 173 nM) 11 different leukemia cancer cell lines. However, THAL-SNS-032 was less potent than the selective CDK9 inhibitor NVP-2 (IC_50_ = 9 nM). The anti-proliferative activity of THAL-SNS-032 was nearly 100 times weaker in CRBN negative cells than in CRBN positive cells, while CDK9 inhibitors activity was independent of CRBN status.

The in-vivo ability to degrade CDK9 via PROTAC molecules was examined by Qiu et al. who introduced PROTACs based on Pomalidomide and a selective CDK9 inhibitor, BAY-1143572. In their study, PROTAC B03 showed 20-fold stronger anti-proliferative activity in MV4-11 cells than BAY-1143572 alone, resulting in strong cancer cell inhibition in BALB/c nude mice bearing MV4-11 xenograft [97].

CDK9 degraders show prolonged pharmacodynamic effects compared to CDK9 inhibitions and high on-target selectivity. They can contribute to achieving an irreversible inhibition via CDK9 degradation, overcoming treatment resistance caused by target mutation and limiting effects of off-target toxicity [98]. However, the lack of clinical trials with CDK9 degraders in multiple myeloma makes the final comparison of these methods a matter of the future.

### Perspectives and limitations

CDK9 inhibitors showed broad anti-cancer activity in-vivo and in-vitro, but the results from clinical trials are still uncertain. The first generation of CDK9 inhibitors (Flavopiridol and Seliciclib) targets multiple CDKs and acts in an ATP-competitive manner, which was the main reason for their off-target toxicity and lack of clinical relevance [16, 57]. Novel CDK9 inhibitors are designed to improve tolerance and compliance for patients undergoing treatment [91]. Their systemic toxicity, cytotoxicity to peripheral blood [36] and adverse effects rate [58] seems to be acceptable.

The use of CDK9 inhibitors as a standalone medication is not supported by much evidence. Despite the encouraging results of preclinical studies, their efficacy in clinical trials was mediocre. Only dinaciclib showed encouraging results as a single agent in patients with relapsed multiple myeloma [57]. Flavopiridol showed no anti-MM activity in patients, while the activity of SNS-032 in phase I clinical trial was limited [63, 67]. Although Dinaciclib and RGB-286638 were able to either achieve prolonged remission or stabilize the disease due to their lack of selectivity there is no certainty that this effect was caused by the inhibition of CDK9 [53, 57]. Nevertheless, the combination of CDK9 inhibitors with either Bortezomib, Doxorubicin or Venetoclax seems to overcome resistance to therapy and cause increased apoptosis of MM cells. This effect was observed in most preclinical studies of examined drugs and was later confirmed in clinical trials with AT-7519 and is currently examined in MM patients treated with Dinaciclib, Bortezomib and Dexamethasone (NCT01096342) [49]. CDK9 inhibitors have an established mechanism of synergy with numerous drugs (Table 5). In this scenation CDK9 inhibitors are used as co-agent, the use of more selective inhibitors may reduce systemic toxicity and alleviate resistance to therapy.

CDK9 degraders appeared because of the inability to induce selective CDK9 inhibition [96, 97]. THAL-SNS-032 and PROTAC B03 induced rapid degradation of CDK9 without affecting the levels of other CDKs [94, 97]. Both of them showed higher potency than CDK9 inhibitors, but their activity seems to depend on the expression of CRBN-mediated genes. THAL-SNS-032 was nearly 100 times weaker in CRBN negative cells, while CDK9 inhibitors work independently of CRBN status [94]. CDK9 degraders show prolonged pharmacodynamic effects compared to CDK9 [98]. This is a major advantage over older CDK9 inhibitors, which requires longer and repetitive infusions [90]. Novel, orally active CDK inhibitors, such as voruciclib, started to emerge to improve the compliance with patients, but were not tested in MM treatment yet [91].

Noteworthy, CDK9 inhibitors were tested only in pretreated patients with relapsed/refractory multiple myeloma or unfavorable cytogenetics. Even in those disadvantageous settings their clinical effect in lifting resistance was noticeable. [49] Furthermore, CDK9 inhibitors act independently of p53, causing MM cell apoptosis even in p53-mutated cell lines [47]. P53 target genes function through CDK9-mediated transcription, while CDK9 inhibition can downregulate p53 transcription or increase the concentration of p53. The outcome depends on the degree of CDK9 blockade, which can be overrun by residual CDK9 activity [10, 23]. Moreover, CDK9 inhibitors can restore p53 tumor-suppressing functions by downregulating iASPP [25]. The nuances of p53 and CDK9 interactions are not clearly explained yet, but opens a new perspective for the treatment of patients with loss of p53 function (Fig. 2).

The last decade has significantly improved the understanding of CDK9 and MM biology. While the first generation of CDK9 inhibitors turned out to be lacking as single agents, they seem to potentiate the efficacy of other therapeutics [84, 93]. More selective inhibitors are less toxic and are usually well-tolerated [15]. The mechanism of synergy between CDK9 inhibitors and Bortezomib, Doxorubicin or Venetoclax is established and prompts the incorporation of CDK9 inhibitors into current drug regimens in further clinical trials (Table 5). However, the need for a long drug infusion or the lack of pharmacokinetic data are still obstacles that need to be addressed.

## Data Availability

Not applicable.
